# Coefficient-Shuffled Variable Block Compressed Sensing for Medical Image Compression in Telemedicine Systems

**DOI:** 10.3390/bioengineering11111101

**Published:** 2024-10-31

**Authors:** R Monika, Samiappan Dhanalakshmi, Narayanamoorthi Rajamanickam, Amr Yousef, Roobaea Alroobaea

**Affiliations:** 1 Department of ECE, Faculty of Engineering and Technology, College of Engineering and Technology, SRM Institute of Science and Technology, Chengalpattu District, Kattankulathur 603203, Tamilnadu, India; monikar@srmist.edu.in; 2 Department of Electrical and Electronics Engineering, Faculty of Engineering and Technology, College of Engineering and Technology, SRM Institute of Science and Technology, Chengalpattu District, Kattankulathur 603203, Tamilnadu, India; narayanr@srmist.edu.in; 3 Electrical Engineering Department, University of Business and Technology, Jeddah 23435, Saudi Arabia; 4 Engineering Mathematics Department, Faculty of Engineering, Alexandria University, Alexandria 21544, Egypt; 5 Department of Computer Science, College of Computers and Information Technology, Taif University, Taif 21944, Saudi Arabia; r.robai@tu.edu.sa

**Keywords:** medical imaging, compressive sensing, coefficient shuffling, block compressive sensing, telemedicine

## Abstract

Medical professionals primarily utilize medical images to detect anomalies within the interior structures and essential organs concealed by the skeletal and dermal layers. The primary purpose of medical imaging is to extract image features for the diagnosis of medical conditions. The processing of these images is indispensable for evaluating a patient’s health. However, when monitoring patients over extended periods using specific medical imaging technologies, a substantial volume of data accumulates daily. Consequently, there arises a necessity to compress these data in order to remove duplicates and speed up the process of acquiring data, making it appropriate for effective analysis and transmission. Compressed Sensing (CS) has recently gained widespread acceptance for rapidly compressing images with a reduced number of samples. Ensuring high-quality image reconstruction using conventional CS and block-based CS (BCS) poses a significant challenge since they rely on randomly selected samples. This challenge can be surmounted by adopting a variable BCS approach that selectively samples from diverse regions within an image. In this context, this paper introduces a novel CS method that uses an energy matrix, namely coefficient shuffling variable BCS (CSEM-VBCS), tailored for compressing a variety of medical images with balanced sparsity, thereby achieving a substantial compression ratio and good reconstruction quality. The results of experimental evaluations underscore a remarkable enhancement in the performance metrics of the proposed method when compared to contemporary state-of-the-art techniques. Unlike other approaches, CSEM-VBCS uses coefficient shuffling to prioritize regions of interest, allowing for more effective compression without compromising image quality. This strategy is especially useful in telemedicine, where bandwidth constraints often limit the transmission of high-resolution medical images. By ensuring faster data acquisition and reduced redundancy, CSEM-VBCS significantly enhances the efficiency of remote patient monitoring and diagnosis.

## 1. Introduction

In the modern era, with a growing global population, individuals are facing health challenges influenced by societal pressures and dietary habits. Many have adopted sedentary lifestyles, leading to deteriorating dietary conditions. Consequently, this trend has contributed to the rise in both chronic and infectious diseases. Leveraging technological advancements for early diagnosis has become crucial in improving patient outcomes and potentially saving lives. One such technological approach involves the use of medical imaging to diagnose conditions early and treat them [1]. A variety of medical modalities are employed for diagnostic and therapeutic purposes, including computerized tomography (CT) scans, Magnetic Resonance Imaging (MRI), microscopic imaging, ultrasound imaging, and X-ray imaging [2]. These tools play a pivotal role in the field of healthcare, enabling early detection and intervention [3].

### 1.1. Necessity of Compressing Medical Images

Quality and minimal artifacts in the reconstruction of medical images are essential, as clinical decisions heavily rely on their clarity. Compression algorithms can reduce the number of images taken during a scan while still retaining the necessary diagnostic information. By optimizing the use of radiation and reducing the number of images acquired, the overall radiation dose that the patient receives can be minimized. This is crucial for patients’ safety, particularly when repeated scans are needed. Ultrasound image compression is necessary due to the formation of numerous sequential waves for each image over a long period of time, which limits patient movement. Microscopic imaging requires managing thousands of images per investigation, necessitating compression to manage the extensive data.

Medical image compression can occur at two stages: the measurement stage and the post-acquisition stage [4]. During the measurement stage, faster data acquisition is crucial, especially for imaging modalities that expose patients to harmful radiation for prolonged durations. Compression can assist by selecting a limited number of samples while achieving superior reconstruction quality with these fewer samples. In the post-acquisition stage, the acquired data should be compressed to save space and reduce costs [5,6]. Furthermore, there are instances where medical practitioners may be located remotely. Transmitting medical images wirelessly, either within limited-bandwidth channels or over the internet, is often necessary to ensure uninterrupted diagnosis and treatment. Additionally, the field of medical imaging generates substantial data, incurring associated costs. Reducing costs and obtaining superior-quality recovery of clinical data has led to a growing interest in comprehensive compression techniques. This paper’s objective is to reduce post-acquisition dataset size through the application of compression techniques.

### 1.2. Compressive Sensing for Compressing Medical Images

The compressed sensing (CS) technique enables efficient capture and compression of information from sparse signals into a minimal amount of data [7]. This technique employs optimized reconstruction algorithms to restore the complete data from this condensed information. In contrast, compression algorithms not based on CS face complexities in the data minimization process and are unable to achieve a high compression rate compared to that attained by CS. Additionally, non-CS compression methods are more susceptible to errors, leading to a deterioration in signal quality. CS works better for medical image compression than other methods because it takes advantage of the inherent sparsity found in many medical images [8,9].

However, conventional CS randomly chooses samples from the entire image, leading to suboptimal reconstruction in some regions of the image. In BCS, the image is partitioned into uniform, small parts to ensure an equitable selection of samples across all areas. Nevertheless, a fixed number of samples is selected from each image region, leading to distortions in the blocks.

In the VBCS, the challenge is tackled by introducing variability in sample selection across distinct image areas. This approach enhances the overall quality of the reconstructed image, yet some regions still display blocking artifacts or distortions. To eliminate these distortions, a strategy involves amalgamating frequency coefficients from all blocks, promoting sparsity distribution throughout the entire image [10]. Given its relevance to medical images, the imperative of minimizing the selected samples persists.

### 1.3. Literary Works

The authors in [11] discuss the potential of CS to revolutionize medical imaging by significantly reducing acquisition time, radiation dose, and data storage requirements across modalities like X-ray computed tomography (CT), magnetic resonance imaging (MRI), and ultrasound. This broad perspective is supported by specific studies demonstrating CS’s efficacy. For instance, ref. [12] assessed the impact of CS on MRI acquisition, highlighting improvements in image quality and reduced scan times in clinical settings, thus aiding radiologists and MRI technologists. The practical benefits of CS in MRI are further emphasized in [13], which advocates for its use in pediatric MRI protocols to enhance patient compliance and reduce motion artifacts, ultimately improving diagnostic accuracy and patient experience.

In the realm of biomedical signal compression, ref. [14] explores CS’s application to manage large storage needs, such as compressing EEG signals and DICOM images. This study shows that CS can achieve a compression ratio of 50:1 for medical images, facilitating efficient storage and transmission without compromising diagnostic integrity.

CS’s utility extends to managing healthcare data for specific conditions, as shown in [15], where CS techniques help detect COVID-19 pneumonia by minimizing CT scan image sizes while preserving critical diagnostic regions. Similarly, ref. [16] presents a CS technique for efficient storage and analysis of ECG signals in cloud-based healthcare systems, using the curvelet transform to enhance signal sparsity and manage large data volumes.

Addressing the challenge of long acquisition times in Diffusion Spectrum Imaging (DSI), ref. [17] demonstrates that CS can reduce scan times by up to 80%, maintaining high accuracy and reliability, particularly in well-segmented white matter bundles. This finding was validated in a study involving 26 participants scanned across multiple sessions.

Ref. [18] evaluates the diagnostic efficiency of CS-MRI for rotator cuff tears, showing that CS-MRI significantly improves sensitivity, specificity, and accuracy compared to clinical signs, thus providing high-resolution imaging crucial for accurate diagnosis. Advances in physiological signal acquisition and processing have enhanced disease detection and diagnosis. Ref. [19] reviews integrating compressed sensing (CS) with physiological signals like EEG, ECG, EMG, and EDA, discussing the benefits and drawbacks of CS and its hardware suitability [20].

### 1.4. Research Gap

1. CS approaches are minimally suited for specific imaging modalities such as ultrasound, demanding unique adaptive solutions to increase diagnostic accuracy and data integrity.
2. Limited research exists on minimizing CS measurements for real-time transmission of medical images in low-bandwidth telemedicine settings, which is critical for enhancing remote healthcare delivery.
3. Maintaining high reconstruction quality in motion-sensitive and paediatric imaging remains difficult, since existing CS approaches lack the robustness required for these dynamic environments.

### 1.5. Intent of the Research

1. Employing the proposed VBCS, a modified version of CS, to compress medical images at high compression ratios.
2. Achieving high-precision medical image recovery while preserving fine details.
3. Assisting medical practitioners in storing extensive amount of medical data by lowering storage demands.

### 1.6. Structure and Contributions of This Paper

We propose a coefficient-shuffled energy matrix-based VBCS (CSEM-VBCS) for compressing medical images. It can be employed in two steps. The first step involves designing a measurement matrix that holds the energy values of the coefficients along the leading diagonal, aiding in the selection of signal components with high energy. The second step involves shuffling signal coefficients across the entire image block to help effectively reconstruct all regions of the image. This certainly removes blurs and blocking artifacts across the image blocks. The CSEM-VBCS method presents a novel approach by integrating an innovative energy matrix for selective sampling, significantly improving reconstruction quality and compression ratios compared to existing techniques. This advancement uniquely addresses critical challenges in medical image compression, enhancing both the efficiency and effectiveness of data transmission in clinical settings. Additionally, the method’s adaptability across diverse imaging modalities sets it apart from contemporary methods.

This article presents an overview of the CS and BCS in Section 2, followed by a discussion on the significance of our approach to medical image compression in Section 3. Section 4 presents the results and discussion. Finally, Section 5 provides a summary of the conclusions and outlines future research directions.

## 2. Preliminary Works

An overview of CS and BCS is given in this section.

### 2.1. Compressed Sensing

CS is represented by the following equation:

(1) Y=Φ×X

where *Y* denotes the compressed measurements, Φ is the sensing matrix, and *X* is the original signal. CS aims to recover sparse signals from fewer measurements.

### 2.2. Block Compressed Sensing

BCS is represented using the following equation:

(2) $$y_{b}=\phi_{b}\times x_{b}$$

where $y_{b}$ denotes the compressed measurements for a block, $\phi_{b}$ is the block sensing matrix, and $x_{b}$ is the original block measurements. BCS aims to recover the sparse signals of a block from fewer measurements.

### 2.3. Modified Fast Haar Wavelet Transform (MFWT)

The MFWT is an enhanced version of the traditional Haar Wavelet Transform, offering improved computational efficiency. It utilizes a recursive algorithm to compute wavelet coefficients by iteratively applying the Haar transform.

The MFWT equations can be represented as follows:

(3) $$a_{j,k}=\frac{1}{\sqrt{2}}\left(a_{j+1,2k}+a_{j+1,2k+1}\right)$$

(4) $$d_{j,k}=\frac{1}{\sqrt{2}}\left(a_{j+1,2k}-a_{j+1,2k+1}\right)$$

Here, $a_{j,k}$ represents the approximation coefficient at level *j* and position *k*, while $d_{j,k}$ represents the detail coefficient. The MFWT efficiently computes these coefficients by recursively decomposing the signal.

## 3. Proposed Coefficient-Shuffled Energy Matrix-Based VBCS (CSEM-VBCS)

The proposed CSEM-VBCS method consists of two main steps. First, it involves designing an energy-based measurement matrix. Second, it involves shuffling the coefficients throughout the image blocks to achieve sparsity balance.

### 3.1. Energy Matrix Design

The energy calculation defines the intensity and frequency characteristics of an image. By calculating the energy either in the spatial domain or the frequency domain, one can focus on the most significant components of the image for various image-processing tasks, such as compression, denoising, and feature extraction. The energy can be calculated using the following formula:

(5) $$E=\sum_{u=0}^{m-1}\sum_{v=0}^{n-1}{\left|x_{b}\left[u,v\right]\right|}^{2}$$

Here, $x_{b}\left[u,v\right]$ are the coefficients of the image block after applying MFWT, m and n are the dimensions of the image block, and *u* and *v* are the frequency indices in the horizontal and vertical directions, respectively. After calculating the energy values for each block, these values are used to determine the overall energy proportion (EP) across all blocks using the following equation:

(6) $$EP_{b}=\frac{E(x_{b})}{\sum_{b=1}^{n}E\left(x_{b}\right)}$$

where ’*n*’ is the total number of blocks in the image and ’*b*’ represents the block number. This $EP_{b}$ is used to fix the variable measurement count to each block using the following equation:

(7) $$VM_{i}=EP_{b}\left(TIS-nFS_{i}\right)$$

where

(8) $$TIS=S\times B^{2}\times n$$

’*S*’ represents the sampling rate and $B^{2}$ represents the block size.

(9) $$FS_{i}=\frac{W\times S\times TIS}{n}$$

where ‘*W*’ is the constant sampling rate assignment parameter. A ’*W*’ value of 0.5 is used because a very low ’*W*’ value indicates total adaptivity, while a very high ’*W*’ value indicates traditional CS. The total variable measurement count for each block is set using the following equation:

(10) $$TS_{i}=FS_{i}+VM_{i}$$

The $TS_{i}$ value determines the total number of rows in the energy measurement matrix.

### 3.2. Coefficient-Shuffled Variable Block Compressed Sensing

The transformed coefficients are shuffled randomly to equalize the sparsity throughout all the blocks. The coefficients associated with similar frequency within a block are combined to form a vector $VC_{j}$:

(11) $$VC_{j}=TC_{i}^{j},i=1,2,\cdots N,j=1,2,\ldots B^{2}$$

where $TC_{i}^{j}$ refers to the *i*th block coefficient associated with the *j*th frequency. All the transformed coefficients related to same frequency are shuffled using the following equation:

(12) $$CP_{j}=P\left(VC_{j}\right),j=1,2,\ldots B^{2}$$

To restore the vector, the shuffled coefficients are scattered throughout all the blocks. The restored vector holds new coefficients belonging to the same frequency:

(13) $$RB_{i}=CP_{j}^{i},j=1,2,\ldots B^{2},i=1,2,\ldots N$$

where $CP_{j}$ is the shuffled jth frequency coefficient. The VBCS is then performed using the designed energy matrix using the following equation:

(14) $$y_{b}=\sum_{b=1}^{n}\phi_{vb}x_{b}$$

where $\Phi_{vb}$ is the designed energy matrix. Then, recovery is performed using TOMP algorithm. TOMP modifies the standard OMP approach by incorporating a thresholding step that helps in enhancing the efficiency and accuracy of the sparse recovery process. Instead of selecting a single atom at each iteration as in the case of OMP, TOMP selects all atoms whose correlation with the current residual exceeds a predefined threshold. This can lead to selecting multiple atoms at each iteration, thus potentially speeding up the algorithm’s convergence. The coefficients recovered by TOMP are then regrouped:

(15) $$\hat{VC_{j}}=\hat{D_{i}^{j}},i=1,2,3,\ldots N,j=1,2,\ldots B^{2}$$

where $\hat{D_{i}^{j}}$ is ith recovered vector’s jth coefficient. Coefficient inverse shuffling is then performed using the following equation:

(16) $$\hat{CP_{j}}=P\left(\hat{VC_{j}}\right),j=1,2,\ldots B^{2}$$

The coefficients are rebuilt for the recovered image block as follows:

(17) $$\hat{C_{i}}=\hat{CP_{j}^{i}},j=1,2,3,\ldots B^{2},i=1,2,\ldots N$$

where $\hat{CP_{j}^{i}}$ is the inverse coefficient-shuffled vectors.

## 4. Results

MATLAB R2024a was employed for the simulation. The measurement matrix was a sparse binary random matrix. It was entirely random, leading to results that are averaged over five trials. The image block size was chosen as 8 × 8. Medical images were obtained from the database provided in [21] and simulated for sampling rates ranging from 0.1 to 0.5. The database comprises approximately 3000–5000 medical images of different types. The proposed CSEM-VBCS was tested on 400 medical images (approximately 50–60 images of all type), and the results of a few images are presented below.

### 4.1. Parameters Selection

If the size of ′*B* × *B*′ is too small, there will be a greater number of blocks, leading to complicated computations. If the size of ′*B* × *B*′ is too large, then the block’s characteristics cannot be effectively captured. Optimal results are obtained for a block size of 8 × 8. Hence, ′8 × 8′ is used as the block size. Sampling rates of S = 0.1, 0.3, and 0.5 were chosen, as higher S values will not justify the use of compressed sensing.

### 4.2. Objective Results

The simulation parameters that are considered for the objective result comparison are PSNR, NCC, NAE, and space-saving. Δ values are calculated as the difference between the proposed and existing methods.

From Table 1, it is observed that the proposed CSEM-VBCS method consistently outperforms other compressed sensing techniques in medical image reconstruction, achieving the highest PSNR across various image types and sampling rates. This indicates its superior capability in preserving image quality, particularly at lower sampling rates, where it shows substantial improvements. The proposed method achieves a PSNR increase of 1–3 dB at higher sampling rates and 2–4 dB at lower sampling rates.

Normalized cross-correlation (NCC) has a maximum value of one, indicating a perfect match between two images. Higher NCC values indicate a closer resemblance of the reconstructed image to the original. Conversely, normalized absolute error (NAE) values closer to zero signify a more accurate reconstruction of an image, reflecting minimal absolute error between predicted and actual pixel values. The proposed CSEM-VBCS method consistently shows competitive performance in terms of both NCC and NAE metrics across varying sampling rates, which is evident from Table 2. The proposed CSEM-VBCS method consistently outperforms other methods (BCS, MB-RACS, CMT-ABCS) across different test images and sampling ratios (S = 0.1, 0.3, 0.5). It achieves the highest NCC values (closer to 1) and the lowest NAE values (closer to 0), indicating superior correlation with the original image and lower error rates. The percentage of samples (%S) is calculated using the following formula:

(18) $$\%\;S^{\prime }s=\frac{Chosen\_S^{\prime }s}{Total\_S^{\prime }s}\times 100$$

where *Chosen_S’s* refers to the selected samples, and *Total_S’s* refers to the total samples in the image.

Table 3 compares the sample count and compressed image size for the tooth X-ray image (800 × 400) at various sampling rates. The proposed CSEM-VBCS method is the most efficient, requiring the lowest percentage of samples (10.34% to 24.93%) and achieving the smallest compressed image sizes (94,304 to 73,216 bytes). MB-RACS and CMT-ABCS provide a balance between sample percentage and compressed image size but are less efficient than CSEM-VBCS. The space saving and runtime analyses are shown in Table 4.

Table 4 compares the space-saving efficiency and runtime at different sampling rates. The proposed CSEM-VBCS algorithm demonstrates superior space-saving capabilities, achieving the highest reductions (70.53% to 77.12%) while also exhibiting the fastest runtimes (20.34 to 23.89 s). MB-RACS performs well in terms of space saving (68.39% to 74.47%) but requires a longer runtime (30.75 to 35.12 s).

### 4.3. Subjective Evaluation

**Figure 1:**
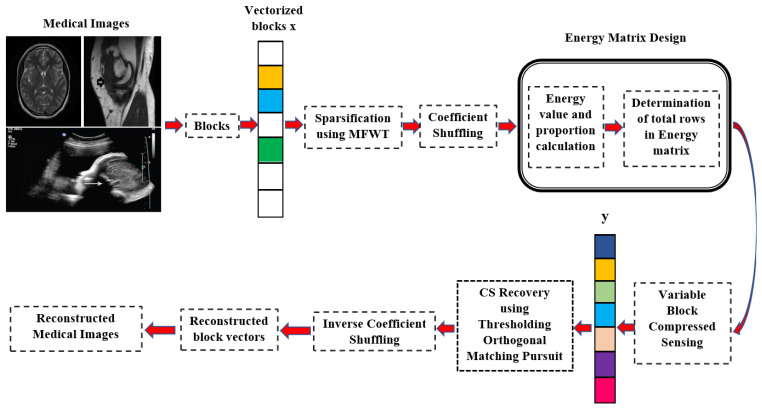
Block diagram of the proposed CSEM-VBCS.

Figure 1 presents a subjective quality comparison of the reconstructed medical images using the proposed algorithm and other algorithms from the literature at a sampling rate (SR) of 0.1. In the results, the yellow boxes highlight blocking artifacts, while the red boxes indicate improper block reconstructions. The proposed CSEM-VBCS method demonstrates superior subjective quality compared to the other methods. Blocking artifacts and improper block reconstructions are notably reduced.

**Figure 2:**
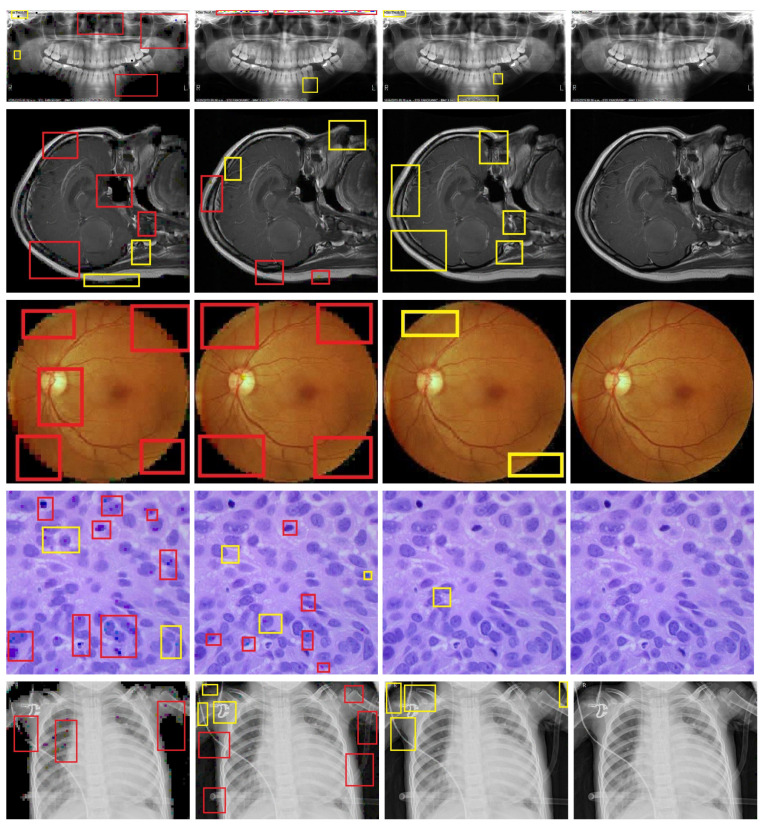
Subjective quality assessment at SR = 0.1. Column 1 corresponds to the original image, and columns 2, 3, and 4 are reconstructed using BCS, MB-RACS, CMT-ABCS, and the proposed CSEM-VBCS, respectively. Blocking artifacts and improper block reconstructions are marked with yellow and red boxes, respectively.

Figure 1 clearly demonstrates that the images reconstructed using the proposed method retain a high clarity, free from blocking artifacts and improper block reconstructions. In contrast, the images reconstructed using other methods exhibit these issues. This highlights the effectiveness of the proposed method in producing higher-quality reconstructions. The yellow boxes highlight blocking artifacts, indicating visual distortions at block boundaries, while the red boxes mark regions with improper block reconstructions, showing areas where image details are inaccurately reconstructed. To analyze the clarity of the reconstructed images, the results were magnified, with the magnified sections displayed in Figure 2. Blue boxes are drawn around specific portions of the images to indicate the areas that have been magnified after reconstruction. This approach assesses the effectiveness of the proposed method in preserving image quality and reducing issues such as blocking artifacts and improper block reconstructions.

Based on the magnified results, it is evident that the various medical images compressed using the proposed CSEM-VBCS method maintained high clarity, even at a sampling rate (SR) of 0.3. Minute details remained intact after reconstruction, which is crucial for medical practitioners conducting medical analyses. The images presented were reconstructed using approximately 10% of the samples from the entire image, demonstrating the effectiveness of the CSEM-VBCS method in preserving image quality with minimal data.

### 4.4. Subjective Evaluation Based on Mean Opinion Score (MOS)

Mean opinion score (MOS) is preferred for quality evaluation. It rates the quality of reconstructed images on a scale from 1 to 5, where 1 indicates ’annoying quality’, 2 denotes ’poor quality’, 3 signifies ’fair quality’, 4 indicates ’good quality’, and 5 signifies ’excellent quality’. Fifty images were chosen from the dataset, and the proposed method was used to compress them. The visual quality parameters used for evaluation are categorized into low-level and high-level attributes. Low-level attributes include intensity and variation, while high-level attributes encompass precision, originality, graininess, and coarseness. To achieve ratings, 20 individuals were consulted, comprising 2 PhD experts in image processing and 18 members of the general public. They were briefed on the scoring process and shown both the original input image and its reconstructed counterpart. Their scores were aggregated for evaluation purposes.

Table 5 displays the average MOS scores for different attributes. They have values predominantly above 4. This indicates high perceived quality across these attributes, reflecting positively on the effectiveness of the proposed method employed.

### 4.5. Sparsity Balance Analysis

Sparsity imbalance is the main reason that various ABCS methods produce blocking artifacts, blurs, and block distortions in the reconstructed images. In the images, the sparsity of smooth blocks is higher than that of textured areas, meaning that higher sparsity results in fewer non-zero elements, and vice versa. Each image block contains a varied number of non-zero elements, leading to improper reconstruction in certain areas of the image blocks. Since medical images require high-quality reconstruction with no blurs and imperfections at all sampling rates, sparsity balancing is crucial for achieving perfect reconstruction across all blocks. The sparsity balance analysis for the proposed CSEM-VBCS and other techniques is discussed in this section. The non-zero element counts of certain blocks are listed in Table 6 and the % sparsity is projected in Table 7.

The non-zero count can be used to calculated the % sparsity using the following equation:

(19) $$\%Sparsity=\frac{N-NZ}{N}\times 100$$

where ’*N*’ represents the length of the signal, and ’*NZ*’ is the non-zero element count.

**Figure 3:**
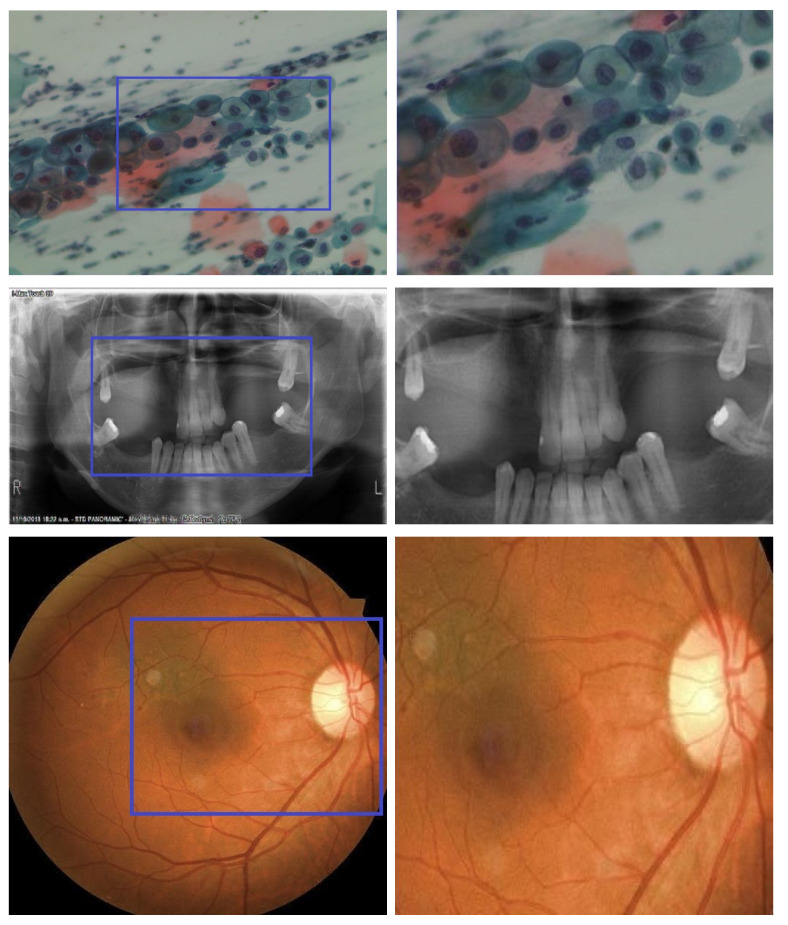

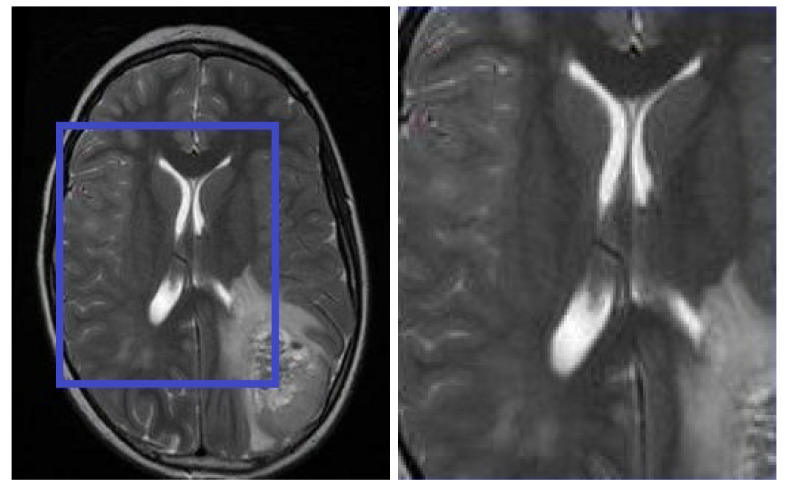
Magnification results after reconstruction at SR = 0.3. Column 1: Reconstructed images using CSEM-VBCS. Column 2: Zoomed images. Blue box represents the zoomed region.

The proposed CSEM-VBCS technique demonstrates high and consistent sparsity values across all blocks, suggesting balanced sparsity throughout the image. This results in visually pleasing reconstruction quality. In contrast, the other techniques exhibit imbalanced sparsity, leading to poorer reconstruction in certain blocks of the image. The sparsity distributions of blocks 1, 3, 5, and 8 are shown in Figure 3.

**Figure 4:**
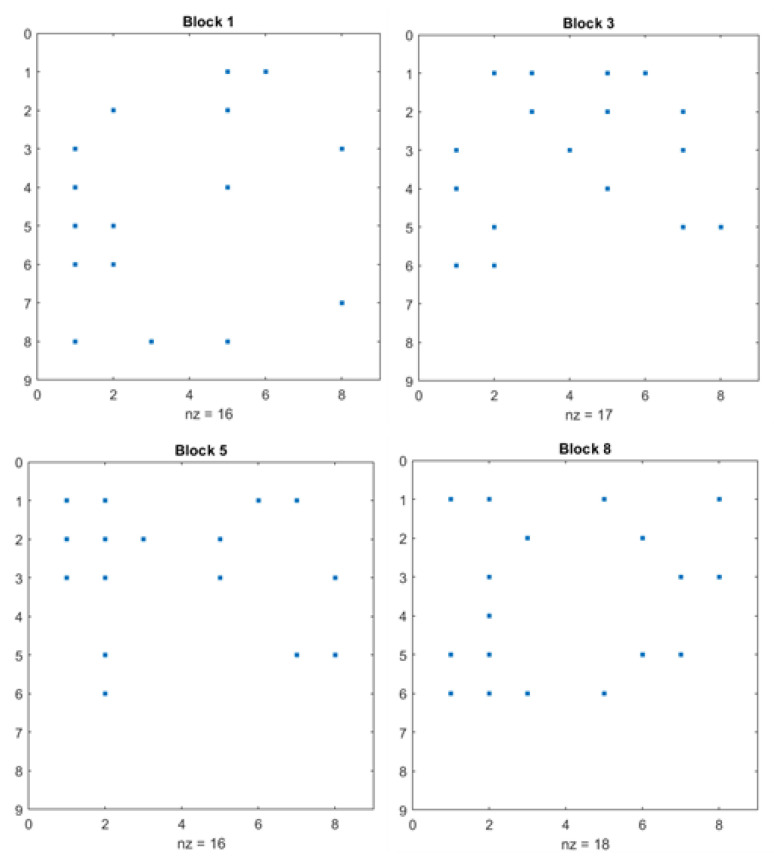
Sparsity distribution of the proposed CS- EM- VBCS for the glaucoma image.

The provided sparsity plot in Figure 4, displays the sparsity pattern of various blocks with 8 × 8 block size. The plot highlights that all four blocks have approximately the same non-zero element count, achieving balance in sparsity across all the blocks. This results in complete elimination of blurs and distortions in the reconstructed image blocks. Almost all areas of the image blocks are reconstructed in an even manner due to even sparsity distribution across all blocks.

## 5. Limitations, Real-World Challenges, and Potential Extensions

- Limitations:
  1. The CSEM-VBCS method requires computationally intensive operations, limiting its use on low-power devices.
  2. Current evaluations are performed under controlled conditions; real-world variability in medical image types could impact performance consistency.
- Real-world challenges:
  1. Integrating CSEM-VBCS into current imaging workflows may necessitate changes to compatibility requirements.
  2. Ensuring data security during image transmission is essential, as compressed medical data may be vulnerable to breaches.
  3. Real-time processing is crucial for clinical use, but achieving this while preserving image quality and compression efficiency is challenging, particularly in resource-constrained environments.
- Potential extensions:
  1. Optimizing CSEM-VBCS for low-power processors would allow its deployment on portable and embedded devices, enhancing its reach in remote diagnostics.
  2. A real-time processing framework could increase the method’s usability in clinical settings, supporting fast, efficient diagnosis and monitoring.
  3. Incorporating machine learning for adaptive energy matrix selection could further boost performance, allowing the method to dynamically adapt to various imaging conditions and improve reconstruction quality.

## 6. Conclusions and Future Work

The proposed Coefficient-Shuffling Variable Block Compressed Sensing (CSEM-VBCS) method introduced in this paper effectively addresses the challenge of high-quality image reconstruction in medical imaging. By utilizing an energy matrix for selective sampling, this approach achieves substantial compression ratios and maintains good reconstruction quality, which are critical for telemedicine and remote diagnostics. The experimental evaluations demonstrate a significant enhancement in performance metrics compared to current state-of-the-art techniques, making CSEM-VBCS a promising solution for the efficient compression and transmission of medical image data, which is crucial for the continuous monitoring and diagnosis of patients. The CSEM-VBCS method addresses challenges in achieving high-quality image reconstruction at substantial compression ratios, ensuring efficient data transmission for continuous patient monitoring. This capability supports telemedicine workflows, allowing for accurate remote diagnostics, even with limited bandwidth. Broader applications in telemedicine workflows could support real-time, high-quality remote assessments, enabling more effective clinical decision-making. Future work can explore the implementation of CSEM-VBCS on low-power processors to assess its efficiency in resource-constrained environments and facilitate adoption in portable diagnostic devices. Additionally, a real-time processing framework for CSEM-VBCS could further improve its practicality in clinical settings. Finally, integrating machine learning algorithms to optimize the energy matrix selection process and dynamically adapt to varying medical image characteristics could yield further improvements in performance.

## Figures and Tables

**Table 1:** PSNR comparison for images of block size (8×8).

TEST IMAGE	BCS	ΔPSNR	MB-RACS	ΔPSNR	CMT-ABCS [4]	ΔPSNR	Proposed CSEM-VBCS
S = 0.1							
Tooth X-ray	23.4034	−17.082	39.1192	−1.3664	39.5743	−0.9113	40.4856
Glaucoma	27.1985	−4.9431	31.0948	−1.0468	31.2945	−0.8471	32.1416
Lung cancer	34.1974	−9.0001	43.1957	−0.0018	43.0975	−0.1	43.1975
MRI	28.9465	−4.27	32.1954	−1.0211	31.4519	−1.7646	33.2165
Pneumonia CT	27.1974	−9.0013	34.1984	−2.0003	35.1974	−1.0013	36.1987
S = 0.3							
Tooth X-ray	25.953	−16.086	40.0424	−1.997	41.2932	−0.7462	42.0394
Glaucoma	29.1685	−4.9263	33.1975	−0.8973	33.1597	−0.9351	34.0948
Lung cancer	37.1974	−8.0011	41.1899	−4.0086	42.2949	−2.9036	45.1985
MRI	29.4926	−5.7049	33.0975	−2.1	34.8946	−0.3029	35.1975
Pneumonia CT	29.9845	−8.777	36.1974	−2.5641	37.1984	−1.5631	38.7615
S = 0.5							
Tooth X-ray	27.0483	−16.99	42.4023	−1.6361	42.234	−1.8044	44.0384
Glaucoma	30.5264	−5.4165	34.5954	−1.3475	34.3729	−1.57	35.9429
Lung cancer	39.1657	−6.9825	44.1954	−1.9528	45.6045	−0.5437	46.1482
MRI	31.8649	−4.2297	34.9561	−1.1385	35.1425	−0.9521	36.0946
Pneumonia CT	32.1974	−7.3026	39.3998	−0.1002	38.1209	−1.3791	39.5000

**Table 2:** NCC and NAE comparison.

Test Image	BCS		MB-RACS		CMT-ABCS [4]		Proposed CSEM-VBCS	
	NCC	NAE	NCC	NAE	NCC	NAE	NCC	NAE
S = 0.1								
Tooth X-ray	0.7623	0.8834	0.9743	0.6176	0.9734	0.7907	0.9985	0.4612
Lung cancer	0.7679	0.7794	0.8623	0.5834	0.9023	0.6298	0.9896	0.4184
Pneumonia CT	0.7679	0.6937	0.7969	0.5532	0.8348	0.4734	0.9789	0.3592
X-ray	0.7434	0.6934	0.9823	0.3134	0.8946	0.4865	0.9779	0.2947
Microscopic	0.7823	0.5025	0.9903	0.4679	0.9912	0.5348	0.9934	0.4375
S = 0.3								
Tooth X-ray	0.8874	0.339	0.9814	0.5965	0.9827	0.7202	0.9997	0.2183
Lung cancer	0.8824	0.4685	0.8868	0.4892	0.9284	0.5965	0.9898	0.2637
Pneumonia CT	0.779	0.4195	0.8036	0.5098	0.8497	0.6134	0.9959	0.2418
X-ray	0.7879	0.4991	0.9877	0.2115	0.9078	0.3991	0.9905	0.1593
Microscopic	0.8931	0.3402	0.9933	0.3478	0.9934	0.418	0.9946	0.2142
S = 0.5								
Tooth X-ray	0.9009	0.3121	0.9893	0.2427	0.8929	0.5012	0.9998	0.1698
Lung cancer	0.9807	0.4532	0.8947	0.3644	0.8955	0.3162	0.9975	0.1872
Pneumonia CT	0.8934	0.3479	0.8821	0.4236	0.8959	0.413	0.9995	0.2134
X-ray	0.9712	0.1768	0.9943	0.1583	0.9981	0.1579	0.9992	0.102
Microscopic	0.9653	0.2447	0.9929	0.3176	0.9979	0.2697	0.9989	0.1623

**Table 3:** Percent samples and compressed image size comparison for tooth X-ray image of block size (8×8).

Image: Tooth X-ray (800 × 400)						
Algorithm	% Samples Chosen			Compressed Image Size (Bytes)		
	S = 0.1	S = 0.3	S = 0.5	S = 0.1	S = 0.3	S = 0.5
BCS	15.87	22.46	30.34	110,944	105,248	100,832
MB-RACS	13.22	21.38	28.35	101,152	95,264	81,696
CMT-ABCS [4]	11.65	20.58	26.85	106,496	99,936	98,784
Proposed CSEM-VBCS	10.34	18.48	24.93	94,304	84,256	73,216

**Table 4:** Percent space saving and runtime comparison for tooth X-ray image of block size (8×8).

Image: Tooth X-ray (800 × 400)						
Algorithm	Space Saving (%)			Runtime (S)		
	S = 0.1	S = 0.3	S = 0.5	S = 0.1	S = 0.3	S = 0.5
BCS	65.33	67.11	68.49	33.74	34.85	39.13
MB-RACS	68.39	70.23	74.47	30.75	32.75	35.12
CMT-ABCS [4]	66.72	68.77	69.13	27.58	29.56	30.45
Proposed CSEM-VBCS	70.53	73.67	77.12	20.34	22.75	23.89

**Table 5:** MOS scores of reconstructed images.

Attribute	Average MOS
Intensity	4.3
Variation	4.3369
Precision	4.5471
Originality	4.5421
Graininess	4.5341
Coarseness	4.435

**Table 6:** Non-zero element count of the techniques.

Image = Tooth X-ray Image; Block Size = 8 × 8, S = 0.1									
Technique	Non-Zero Element Count								
	B1	B2	B3	B4	B5	B6	B7	B8	B9
EABCS	23	21	16	12	10	13	18	25	26
ARWS-ABCS	25	37	33	13	36	23	21	26	11
BCS	13	13	16	24	24	26	31	29	25
MB-RACS	8	13	15	17	23	26	21	27	15
CMT-ABCS [4]	24	25	26	19	12	26	27	21	19
Proposed CSEM-VBCS	16	17	15	‘16	15	15	16	16	17

**Table 7:** Percent sparisty of the techniques.

Image = Tooth X-ray Image; Block Size = 8 × 8, S = 0.1									
Technique	% Sparsity								
	B1	B2	B3	B4	B5	B6	B7	B8	B9
EABCS	64.06	67.19	75.00	81.25	84.38	79.69	71.88	60.94	59.38
ARWS-ABCS	60.94	42.19	48.44	79.69	43.75	64.06	67.19	59.38	82.81
BCS	79.69	79.69	75.00	62.50	62.50	59.38	51.56	54.69	60.94
MB-RACS	87.50	79.69	76.56	73.44	64.06	59.38	67.19	57.81	76.56
CMT-ABCS [4]	62.50	60.94	59.38	70.31	81.25	59.38	57.81	67.19	70.31
Proposed CSEM-VBCS	75.00	73.44	76.56	75.00	76.56	76.56	75.00	75.00	73.44

## Data Availability

The original contributions presented in the study are included in the article, further inquiries can be directed to the corresponding authors.
